# The Galloyl Group Enhances the Inhibitory Activity of Catechins against LPS-Triggered Inflammation in RAW264.7 Cells

**DOI:** 10.3390/foods13162616

**Published:** 2024-08-21

**Authors:** Jinming Peng, Guangwei Chen, Shaoxin Guo, Ziyuan Lin, Jun Li, Wenhua Yang, Gengsheng Xiao, Qin Wang

**Affiliations:** 1 Guangdong Key Laboratory of Science and Technology of Lingnan Specialty Food, Zhongkai University of Agriculture and Engineering, Guangzhou 510225, China; pengjmiyz@163.com (J.P.); linzy20010225@163.com (Z.L.); gshxiao@aliyun.com (G.X.); 2 Key Laboratory of Green Processing and Intelligent Manufacturing of Lingnan Specialty Food, Ministry of Agriculture, Zhongkai University of Agriculture and Engineering, Guangzhou 510225, China

**Keywords:** catechins, galloyl group, inhibitory, inflammation, TLR4/MAPK/NF-κB pathway

## Abstract

The galloyl group in catechins was confirmed to be crucial for their health benefits. However, whether the catechins’ galloyl group had a contribution to their anti-inflammation remains unclear. This study investigated the anti-inflammation properties and mechanisms of catechins in RAW264.7 cells by using ELISA, fluorometry, flow cytometer, Western blot, and molecular docking. Results showed that the galloyl group enhanced the inhibitory abilities of catechins on inflammatory cytokines (NO, PGE_2_, IL-1β, and TNF-α) and ROS release in LPS-induced cells. This suppression was likely mediated by delaying cells from the G0/G1 to the S phase, blocking COX-2 and iNOS via the TLR4/MAPK/NF-κB pathway with PU.1 as an upstream target. The research proved that the existence of galloyl groups in catechins was indispensable for their anti-inflammatory capacities and offered a theoretical basis for the anti-inflammatory mechanism of galloylated catechins. Future research is needed to verify the anti-inflammatory effects of catechins in various sources of macrophages or the Caco-2/RAW264.7 cell co-culture system.

## 1. Introduction

Normally, inflammation facilitates an automatic defense response [1]. Nevertheless, exaggerated inflammation can cause pathological alterations, such as oxidative stress, extracellular matrix remodeling, angiogenesis, and fibrosis [2]. These changes will result in many chronic illnesses, such as type 2 diabetes, angiocardiopathy, and neurodegenerative disorders [3,4]. Pharmacological interference is deemed an effective strategy to control inflammation-associated diseases. Traditional treatments depended mainly on steroid and non-steroidal anti-inflammatory medicines; however, the side effects of both drugs are becoming increasingly commonplace [5]. At present, natural anti-inflammatory ingredients, including polyphenols and polysaccharides, draw broad attention because of their potential applications [6,7].

Lipopolysaccharide (LPS), a principal constituent of the outer membrane of Gram-negative bacteria, robustly triggers inflammatory signaling pathways in macrophages [8]. Inflammation in LPS-induced macrophages is initiated by the interaction of LPS with LPS-binding protein (LBP), and then cluster of differentiation 14 (CD14) transports LPS to the toll-like receptor 4 (TLR4)/myeloid differential protein 2 (MD2) complex [9]. TLR4/MD2 further activates the inflammatory cascade through both the myeloid differentiation primary response 88 (MyD88) and TIR-domain-containing adapter-inducing interferon-β (TRIF) [10]. This event triggers the downstream signaling cascades, especially the nuclear factor-kappa-B (NF-κB) and mitogen-activated protein kinases (MAPKs) pathways, subsequently accelerating the release of inflammatory cytokines (e.g., IL-1β and TNF-α) [11,12].

**Figure 1:**
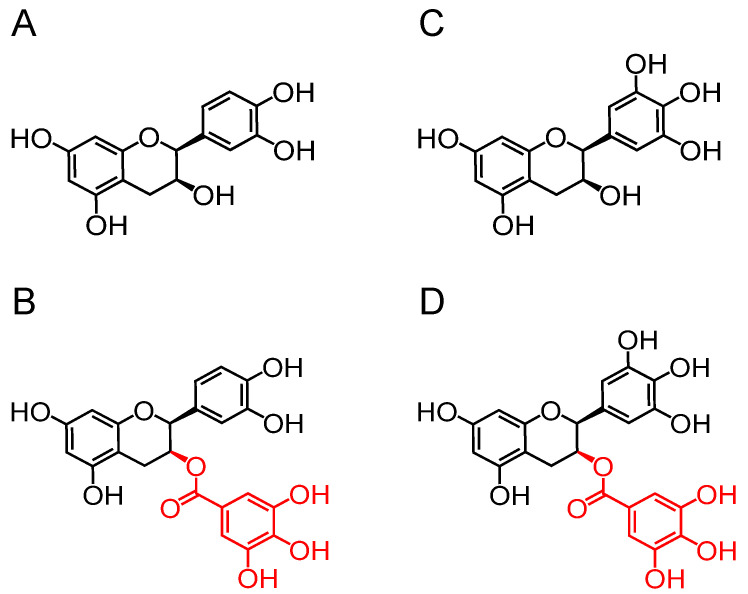
The chemical structures of four catechins: (**A**) EC, epicatechin; (**B**) ECG, epicatechin-3-gallate; (**C**) EGC, epigallocatechin; and (**D**) EGCG, epigallocatechin-3-gallate. The group in red is the galloyl group.

Green tea, made from the *Camelia sinensis* leaves, is a globally cherished natural beverage due to its diverse health-promoting benefits [13]. Green tea is particularly rich in polyphenols, including phenolic acids, flavonoids, procyanidins, etc. [14]. The catechins represent 80–90% of the green tea flavonoids, making up approximately 30% of tea leaves (dry weight) [15]. Tea catechins mainly contain epicatechin (EC) and epigallocatechin (EGC), which have no galloyl group, and epicatechin gallate (ECG) and epigallocatechin gallate (EGCG), which have one galloyl group at their 3-positions (Figure 1). Numerous studies have proved that the galloyl group in catechins played a decisive role in their biological activities, such as antioxidant [16], anti-obesity [17], antiviral [18], and anti-cancer effect [19]. Nevertheless, it has not yet been documented whether the galloyl group augmented the anti-inflammatory properties of catechins. Thus, this research aimed to evaluate the effects of the galloyl group on enhancing the anti-inflammatory capabilities of catechins in LPS-triggered RAW264.7 macrophages. Additionally, the anti-inflammation mechanisms of catechins were investigated by using ELISA, fluorometry, flow cytometer, Western blot, and molecular docking.

## 2. Materials and Methods

### 2.1. Chemicals and Reagents

Catechins were sourced from Yuanye Bio-Technology (Shanghai, China), with a purity of over 98%. Moreover, 3-(4,5-dimethylthiazole-2-yl)-2 and 5-diphenyltetrazolium bromide (MTT) were from Solarbio Science and Technology (Beijing, China). Antibodies were obtained from Cell Signaling Technology (Cambridge, MA, USA). Mouse PGE_2_, IL-1β, and TNF-α ELISA kits and reactive oxygen species (ROSs) detection kits were from Beyotime Biotechnology (Shanghai, China).

### 2.2. Cell Culture and Treatment

RAW264.7 cells were cultivated in 10% fetal bovine serum (FBS)-supplemented DMEM (Gibco, Miami, FL, USA), 100 U/mL of penicillin, and streptomycin at 37 °C with 5% CO_2_. After confluence, the cells were incubated in 10% FBS-supplemented DMEM with 1 μg/mL LPS (Sigma-Aldrich, St. Louis, MO, USA) for 24 h.

### 2.3. Cell Viability Assay

Cells were seeded at a density of 5 × 10^3^ cells/well into a 96-well plate. Upon reaching confluence, the cells were exposed to fresh medium containing 50–150 μM of catechins for 24 h. After that, cells were colored by MTT dye (0.5 mg/mL) for 4 h, and then the absorbance of DMSO-dissolved cells was read at OD_570 nm_.

### 2.4. Determination of Pro-Inflammatory Cytokines

As stated in the earlier reference [20], the measurement of NO in the cell culture medium was conducted by using the Griess method. The medium supernatant was combined with isopyknic Griess reagent, and then measured the absorbance at OD_540 nm_. The level of PGE_2_, IL-1β, and TNF-α in cell culture medium was analyzed by using the corresponding ELISA kit based on the standard procedure.

### 2.5. ROS Assay

Intracellular ROSs were detected as previously mentioned with a slight change [21]. RAW264.7 cells were treated with or without catechins (100 μM) and then marked by DCFH-DA probe (2.5 μM) for 15 min at 37 °C. The fluorescent signal was visualized by an OLYMPUS IX73 microscope (OLYMPUS, Tokyo, Japan), and the fluorescent strength was recorded by emission at 485 nm and excitation at 535 nm.

### 2.6. Cell Cycle Analysis

Quantitative analysis of the cell cycle was performed as previously mentioned [22]. RAW264.7 cells were intervened with or without catechins (100 μM) containing LPS (1 μg/mL) for 24 h. Next, macrophages were collected with PBS and fixed overnight in cold 75% EtOH, and then treated with RNase A (50 μg/mL) and PI (50 μg/mL). Cell cycle analysis was carried out using FACSVerse (BD Biosciences, Franklin Lakes, NJ, USA).

### 2.7. Immunoblot Analysis

Protein expression levels were assessed through immunoblot analysis as published [23]. The antibody details are as follows: rabbit anti-β-actin (GB15003, Servicebio), rabbit anti-MAPKs (#9926, CST), rabbit anti-NF-κB (#8242, CST), rabbit anti-Phospho-MAPKs (#9910, CST), rabbit anti-Phospho-NF-κB (#3033, CST), rabbit anti-COX-2 (GB115672, Servicebio), rabbit anti-iNOS (#2982, CST), rabbit anti-CD14 (#93882, CST), rabbit anti-TLR4 (#14358, CST), rabbit anti-MD-2 (ab24182, Abcam), rabbit anti-MyD88 (#4283, CST), rabbit anti-TRAF6 (#8028, CST), and anti-rabbit IgG (#7074, CST).

### 2.8. Molecular Docking

The molecular docking study was conducted to predict the interactions between PU.1-DNA (PDB ID: 1PUE) and catechins. The protein structure was prepared using Discovery Studio v.4.5, including water molecules deletion, hydrogen atom addition, and energy minimization. Ligands were prepared by using ChemBioDraw v.14.0. Docking was executed using AutoDock Vina v.1.2.0; the top-ranked poses were selected based on the lowest binding energy.

### 2.9. Statistical Analysis

GraphPad Prism 9.0 and SPSS 27.0 were adopted for data visualization and significance analysis. Results were shown as mean ± SD from no less than three experimental replications. Difference significance was analyzed using one-way ANOVA with Tukey’s post-test, with *p* < 0.05 indicating significance.

## 3. Results and Discussion

**Figure 2:**
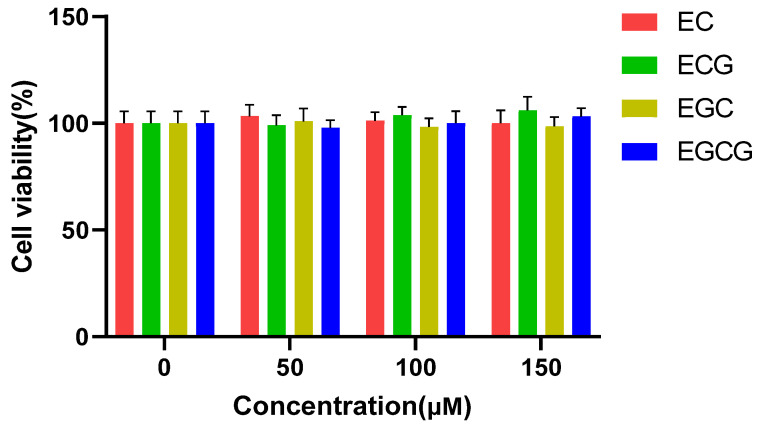
Influences of catechins (EC, epicatechin; ECG, epicatechin-3-gallate; EGC, epigallocatechin; EGCG, epigallocatechin-3-gallate) on RAW 264.7 cell viability. Results were displayed as mean ± SD from no less than three experimental replications.

Prior studies have established that the catechins’ galloyl group augmented their biological activities, for example, anti-oxidative, anti-obesity, and anti-angiogenesis [24]. Nevertheless, the anti-inflammatory properties of galloylated catechins have yet to be elucidated. This study aimed to define the importance of the galloyl moiety for catechins’ anti-inflammatory efficacies. At first, the influences of four catechins on cell viability were measured by MTT colorimetry. Figure 2 presents that all the catechins were found to be non-cytotoxic within 50–150 μM. Therefore, these concentrations were selected for the follow-up study.

**Figure 3:**
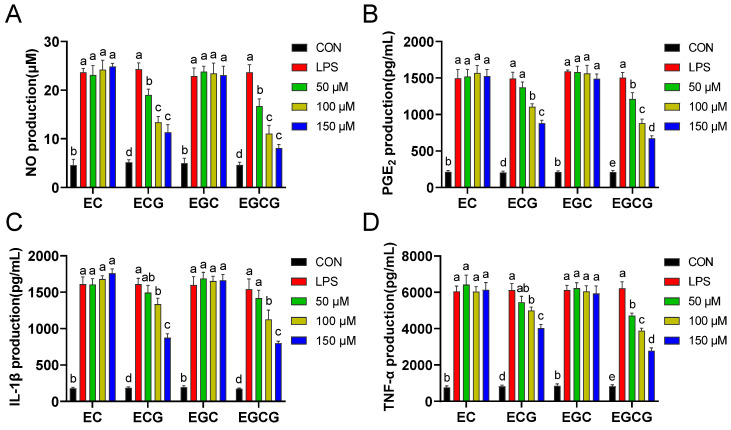
Effects of catechins (EC, epicatechin; ECG, epicatechin-3-gallate; EGC, epigallocatechin; EGCG, epigallocatechin-3-gallate) on the levels of NO (**A**), PGE_2_ (**B**), IL-1β (**C**), and TNF-α (**D**) in RAW264.7 cells. Results were displayed as mean ± SD from no less than three experimental replications. Diverse lowercases in the histogram denote significant differences (*p* < 0.05).

NO, PGE_2_, IL-1β, and TNF-α have been established as pivotal biomarkers of inflammation. The substantial secretion of these cytokines by macrophages can precipitate a myriad of diseases associated with inflammation [25,26]. Decreasing the pro-inflammatory agent levels is recognized as an efficacious strategy for mitigating diverse forms of inflammatory responses [27]. Therefore, the inhibitory activities of different catechins on NO, PGE_2_, IL-1β, and TNF-α release were evaluated in LPS-stimulated macrophages. Figure 3 shows that the levels of these pro-inflammatory factors were significantly (*p* < 0.05) improved after LPS treatment. However, the observed increases were concentration-dependently inhibited by galloylated catechins (ECG and EGCG) but not by non-galloylated catechins (EC and EGC). Yu et al. reported that the inflammatory agents (NO, IL-1β, TNF-α, and IL-6) scavenging abilities of procyanidin B2-3′-O-gallate (with one galloyl moiety) were superior to that of procyanidin B2 (without galloyl moiety) [28]. Thus, these results proved that the catechins’ galloyl group was instrumental in their capacity to exert anti-inflammatory effects.

**Figure 4:**
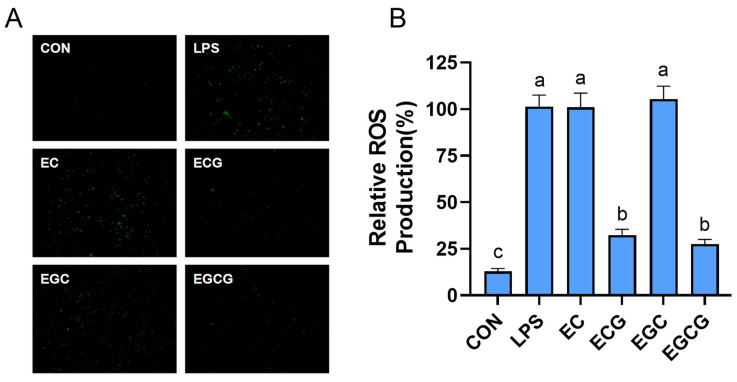
Impacts of catechins (EC, epicatechin; ECG, epicatechin-3-gallate; EGC, epigallocatechin; EGCG, epigallocatechin-3-gallate) on ROS release in LPS-induced RAW264.7 cells. (**A**) Fluorescence microscopy observation. (**B**) Quantitative analysis. The cells were exposed to LPS (1 μg/mL) with or without the addition of catechins (100 µM) for 24 h. Results were displayed as mean ± SD from no less than three experimental replications. Diverse lowercases in the histogram denote significant differences (*p* < 0.05).

Once stimulated, inflammatory cells will release a variety of ROS, which can cause cell and tissue damage [29]. Excess ROS can also activate inflammation-related signaling pathways; thus, eliminating ROS is an effective way to control inflammatory diseases and their complications [30]. This research assessed the inhibition activities of catechins on the generation of ROS in LPS-triggered RAW264.7 cells. Figure 4 shows the ROS fluorescent intensity in LPS-induced cells was obviously higher than that in the non-treated cells (CON). By comparison, ECG and EGCG markedly (*p* < 0.05) reduced the fluorescence intensity, whereas EC and EGC did not exhibit any noticeable quenching effects. The findings indicated that the catechins’ galloyl group was essential for their ROS inhibition abilities. A previous study has confirmed that the free radical scavenging abilities of the galloylated catechins (ECG and EGCG) were superior to those of the non-galloylated molecules [16]. Thus, the elimination ability of free radicals might be a potential mechanism for ECG and EGCG in decreasing ROS.

**Figure 5:**
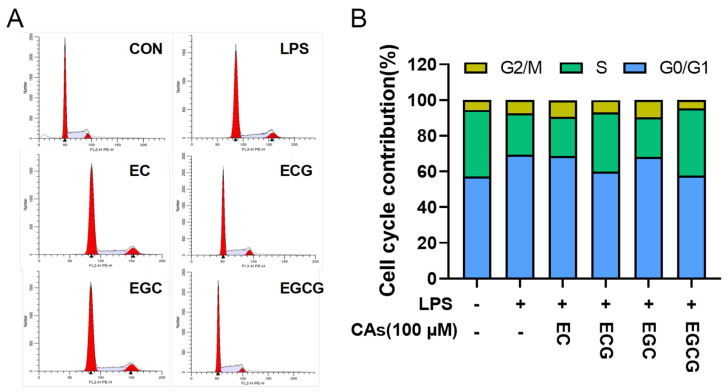
Regulatory influences of catechins (CAs: EC, epicatechin; ECG, epicatechin-3-gallate; EGC, epigallocatechin; EGCG, epigallocatechin-3-gallate) on cell cycle progression. (**A**) RAW264.7 cells were harvested after 24 h of LPS induction, colored with PI, and subsequently detected using flow cytometry. (**B**) The modulation of cell cycle by CAs was represented as the relative proportion of cells in three different phases.

Inflammation usually causes cell growth inhibition and even apoptosis [31]. The cell cycle involves several critical checkpoints, notably the G0/G1 to S and G2 to M phases. The G0/G1 to S transition is recognized as the most critical juncture in the process of DNA replication and mitosis [32]. In the present study, the impacts of four catechins on the cell cycle progression were examined in LPS-stimulated cells. Figure 5 shows that the cells increase in the G0/G1 phase (from 57.28% to 69.33%) and the cells decrease in the S phase (from 37.13% to 23.20%) were observed after LPS induction, suggesting that LPS prevented cells from entering the S phase. However, ECG and EGCG released the LPS-induced G0/G1 phase block, which was aligned with Li et al. (2022), who indicated that the anti-inflammatory activities of dandelion were achieved by relieving macrophage G0/G1 phase [33]. Compared to ECG and EGCG, EC and EGC exhibited minimal impacts. This proved that the galloyl group within catechins was pivotal in mitigating cell cycle arrest in LPS-induced RAW264.7 macrophages.

**Figure 6:**
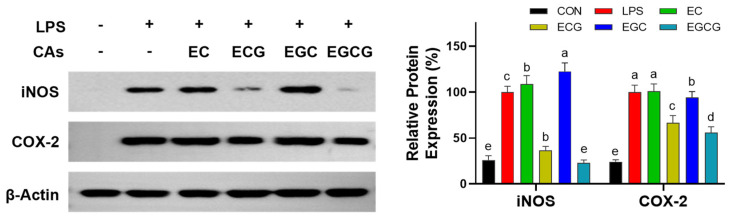
Impacts of catechins (CAs: EC, epicatechin; ECG, epicatechin-3-gallate; EGC, epigallocatechin; EGCG, epigallocatechin-3-gallate) on the iNOS and COX-2 expression in macrophages. Cells were exposed to LPS (1 μg/mL) with or without the addition of catechins (100 µM) for 24 h. Results were displayed as mean ± SD from no less than three experimental replications. Diverse lowercases in the histogram denote significant differences (*p* < 0.05).

Inducible nitric oxide synthase (iNOS) serves as the primary regulator of NO production under pro-inflammatory conditions [34]. Cyclooxygenase-2 (COX-2) is the pro-inflammatory enzyme that mediates the conversion of arachidonic acid to prostaglandin H2, which functions as a precursor for the synthesis of PGE_2_, particularly in activated macrophages [35]. The excessive expression of iNOS and COX-2 can result in elevated levels of pro-inflammatory factors [36]. Therefore, this study further examined the effects of four catechins on the expression levels of iNOS and COX-2. Figure 6 shows that the iNOS and COX-2 expression levels in cells were prominently (*p* < 0.05) increased after LPS treatment. In contrast, ECG and EGCG markedly (*p* < 0.05) restrained these two markers, while no inhibition was found when intervened with EC and EGC. A previous study also proved that prodelphinidin B2 3,3′-di-O-gallate, having two galloyl groups, exhibited the strongest inhibitory ability against COX-2 expression in LPS-activated RAW264 cells, whereas prodelphinidin B2, having no galloyl group, failed to display an inhibition activity [37]. This suggested that the catechins’ galloyl group was vital for their inhibitory abilities on iNOS and COX-2.

**Figure 7:**
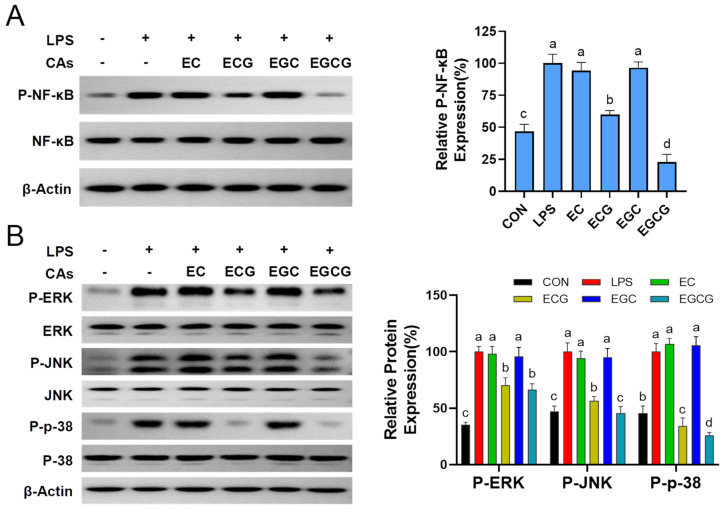
Effects of catechins (CAs: EC, epicatechin; ECG, epicatechin-3-gallate; EGC, epigallocatechin; EGCG, epigallocatechin-3-gallate) on MAPK/NF-κB pathway in LPS-triggered macrophages. (**A**) The NF-κB phosphorylation level in cells. (**B**) The ERK, JNK, and p38 phosphorylation levels in cells. Results were displayed as mean ± SD from no less than three experimental replications. Diverse lowercases in the histogram denote significant differences (*p* < 0.05).

NF-κB, a pivotal transcription factor, which modulates the expression of cell cycle regulatory proteins and pro-inflammatory mediators (iNOS and COX-2) [38]. In normal macrophages, NF-κB exists in the cytoplasm as an NF-κB/IκB complex. Once stimulated by LPS, NF-κB transcribes into the nucleus and triggers the excessive release of inflammation cytokines [39]. To ascertain the necessity of the catechins’ galloyl moiety for their anti-inflammatory activities via the NF-κB pathway, the NF-κB phosphorylation level was examined by using immunoblot. Figure 7A presents that the phosphorylated NF-κB level was prominently (*p* < 0.05) up-regulated after LPS stimulation. It was found that ECG and EGCG could dramatically (*p* < 0.05) inhibit NF-κB phosphorylation, while EC and EGC showed no inhibitory action. Wang et al. also found that ECG and EGCG reduced the secretion of inflammatory factors (IL-6, IL-1β, and TNF-α) in LPS-stimulated dental pulp cells by inhibiting activation of the NF-κB [40]. MAPKs, as a key regulator in the communication between cell surface receptors and nucleus DNA, which can transmit extracellular signals through continuous phosphorylation events in response to extracellular stimuli [41]. Previous research has shown that MAPKs activation can further phosphorylate NF-κB, which regulates inflammation-related downstream signaling [42]. Thus, this study further investigated the influences of four catechins on the phosphorylation of ERK, JNK, and p38 in LPS-induced macrophages. Figure 7B shows that LPS prominently (*p* < 0.05) activated the phosphorylation of ERK, JNK, and p38. However, ECG and EGCG markedly (*p* < 0.05) blocked the activation of ERK, JNK, and p38, whereas EC and EGC exhibited no suppressive effects. Liang et al. reported that EGCG blocked cigarette smoke medium-stimulated ROS and IL-8 production by suppressing ERK, p38 MAPK, and NF-κB signaling pathways in human AC16 cardiomyocytes [43]. Therefore, the above findings elucidated that the catechins’ galloyl moiety was indispensable for blocking MAPK/NF-κB activation in LPS-triggered cells, and the anti-inflammation action of galloylated catechins (ECG and EGCG) was achieved by restraining the MAPK/NF-κB pathway, consequently curbing the secretion of inflammatory cytokines.

**Figure 8:**
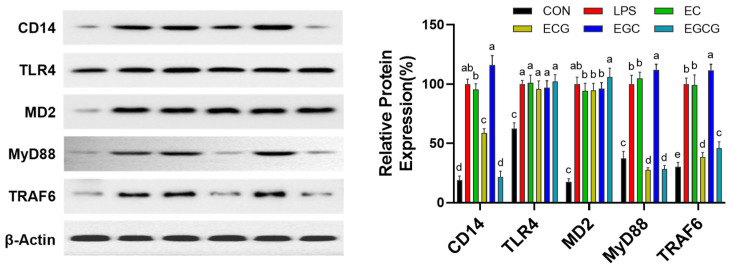
Influences of catechins (CAs: EC, epicatechin; ECG, epicatechin-3-gallate; EGC, epigallocatechin; EGCG, epigallocatechin-3-gallate) on TLR4/CD14 pathway in LPS-triggered RAW264.7 cells. The expression levels of CD14, TLR4, MD-2, MyD88, and TRAF6 in cells. Results were displayed as mean ± SD from no less than three experimental replications. Diverse lowercases in the histogram denote significant differences (*p* < 0.05).

Toll-like receptor 4 (TLR4), an important signaling receptor of LPS, exerts a pivotal influence on the modulation of inflammatory pathways [44]. LPS is conveyed to the TLR4/MD2 complex by CD14; subsequently, TLR4 itself forms a dimer to recruit MyD88 and/or TRIF [45]. For the MyD88-dependent signaling cascade, activated TRAF6 results in the transport, synthesis, and release of inflammatory factors via MAPK/NF-κB signaling cascades [46]. Therefore, this research further evaluated the influences of catechins on the TLR4/CD14 signaling pathway. Figure 8 presents that LPS treatment results in a significant (*p* < 0.05) up-regulation of CD14, TLR4, MD-2, MyD88, and TRAF6 expression levels compared to the untreated cells (CON). ECG and EGCG inhibited CD14 expression without affecting TLR4 and MD2, while EC and EGC showed no inhibitory effects on the three protein expressions. Moreover, ECG and EGCG were found to significantly (*p* < 0.05) attenuate the expression levels of the downstream signaling proteins MyD88 and TRAF6. While EC and EGC had no prominent (*p* > 0.05) inhibiting effects on MyD88 and TRAF6 expressions, and EGC even prominently (*p* < 0.05) up-regulated their expressions. Xue et al. also found that EGCG reduced the levels of inflammatory factors (IL-2, IFN-γ, IL-4, and IL-1) by regulating the TLR4/MyD88/NF-κB signaling pathway to alleviate intestinal inflammation in Sprague–Dawley rats [47]. These results suggested that galloylated catechins (ECG and EGCG) restrain the MAPK/NF-κB signaling cascade by blocking the upstream TLR4/CD14 signaling pathway.

**Figure 9:**
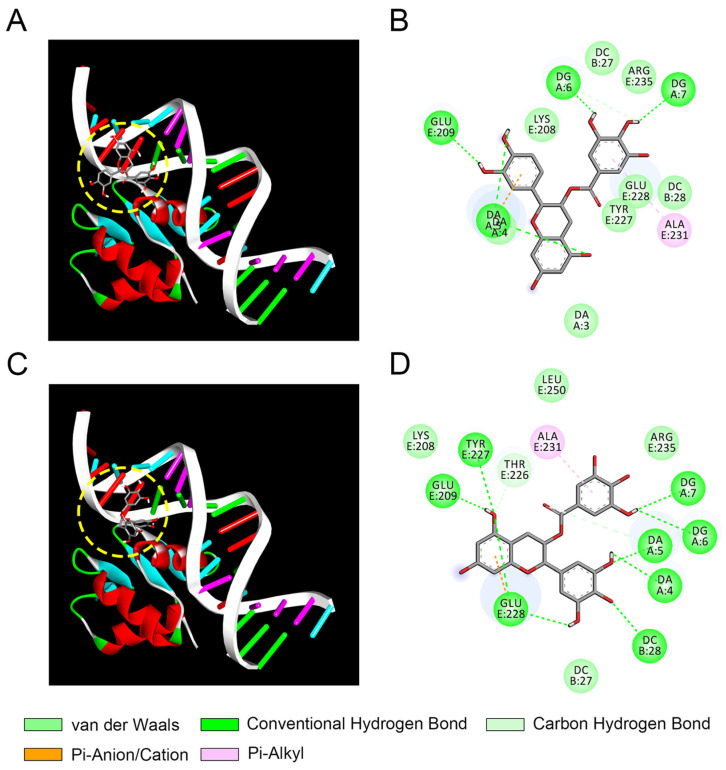
Docking results of the interaction between PU.1 and galloylated catechins (ECG, epicatechin-3-gallate; EGCG, epigallocatechin-3-gallate). (**A**) The 3D (**A**) and 2D (**B**) diagram of ECG-PU.1-DNA interaction. The 3D (**C**) and 2D (**D**) diagrams of EGCG-PU.1-DNA interaction.

The growth and maturation of macrophages are heavily dependent on PU.1, a transcription factor in the E-twenty-six (ETS) family [48]. Previous studies have demonstrated that PU.1 is crucial for regulating TLR1, TLR2, TLR4, and CD14 and the adjustment of TLR signaling by targeting PU.1 [49]. The aforementioned study has demonstrated that ECG and EGCG inhibit the TLR signaling pathway through the down-regulation of CD14 expression. We hypothesized that galloylated catechins (ECG and EGCG) might regulate TLR signaling through interactions with the transcription factor PU.1. Docking analysis was conducted to investigate the binding affinity between catechins and the PU.1-DNA complex. Figure 9A,C shows that ECG and EGCG exhibited different binding poses in the PU.1-DNA complex. The binding free energy of ECG and EGCG with PU.1 was −8.4 and −8.9 kcal/mol, separately. This suggested that EGCG possessed stronger binding capacity with PU.1 than ECG, which was consistent with their anti-inflammatory activity. Figure 9B,D presents that ECG interacted with eight amino acid residues (LYS E:208, TYR E:227, GLU E:228, ARG E:235, DA A:3, DA A:4, DC B:27, and DC B:28) of the PU.1-DNA complex by forming Van der Waals forces; five residues (GLU E:209, DA A:4, DA A:5, DG A:6, and DG A:7) of the PU.1-DNA complex by forming hydrogen bonds; two residues (ALA E:231 and DA A:5) of the PU.1-DNA complex by forming π interactions. EGCG bound to five amino acid residues (LYS E:208, THR E:226, ARG E:235, LEU E:250, and DC B:27) of the PU.1-DNA complex by forming Van der Waals forces; nine residues (DA A:4, DA A:5, DG A:6, DG A:7, DC B:28, GLU: E:209, THR: E:226, TYR: E:227, and GLU: E:228) of the PU.1-DNA complex by forming hydrogen bonds; two residues (GLU E:228 and ALA E:231) of the PU.1-DNA complex by forming π interactions. Xia et al. uncovered that the mechanism of anti-neuroinflammatory activities of sophotokin involved suppression of the TLR4 signaling pathway at the sites of NF-κB and MAPK with PU.1 as a likely upstream target [46]. Therefore, ECG and EGCG might restrain the inflammation-related TLR4 pathway by disturbing the interaction of PU.1 with the DNA.

## 4. Conclusions

To summarize, this study confirmed that the catechins’ galloyl group was indispensable for their anti-inflammatory capacities. This strong anti-inflammatory activity is achieved by disturbing cells from the G0/G1 to the S phase, as well as downstreaming iNOS and COX-2 suppression by blocking the TLR4/MAPK/NF-κB signaling cascade and further restraining the generation of inflammatory agents (NO, PGE_2_, IL-1β, and TNF-α) and ROS. Docking analysis suggested that ECG and EGCG could bind with the PU.1-DNA complex by forming hydrogen bonds, Van der Waals forces, and π interactions, preventing the DNA binding. Therefore, galloylated catechins (ECG and EGCG) could serve as a dietary functional factor for alleviating inflammation and inflammation-associated chronic diseases. Considering various inflammatory parts involve different types of cells, future studies are needed to evaluate galloylated catechins’ anti-inflammatory activities in other sources of macrophages or the Caco-2/RAW264.7 cell co-culture model.

## Data Availability

The original contributions presented in the study are included in the article, further inquiries can be directed to the corresponding author.
